# Relationship Between Subclinical Hypothyroidism and the Risk of Cardiovascular Complications

**DOI:** 10.7759/cureus.33708

**Published:** 2023-01-12

**Authors:** Aashi Kaushik, Manjusha Agrawal

**Affiliations:** 1 Obstetrics and Gynaecology, Jawaharlal Nehru Medical College, Datta Meghe Institute of Medical Sciences, Wardha, IND

**Keywords:** levothyroxine therapy, cardiovascular diseases, cardiovascular complications, thyroid dysfunction, subclinical hypothyroidism

## Abstract

Subclinical hypothyroidism is characterized by raised thyroid-stimulating hormone levels in the presence of normal free thyroxine levels. When free thyroxine levels are normal and subclinical hypothyroidism is present, thyroid-stimulating hormone levels are elevated. The impact of subclinical hypothyroidism on the cardiovascular system has recently garnered attention because it is known that thyroid hormones impact the heart and its vasculature. There is compelling evidence linking subclinical hypothyroidism to increased cardiac risks, including changes in blood pressure and cholesterol. It is unclear whether subclinical hypothyroidism is associated with a higher risk of cardiovascular illnesses. In addition to discussing the advantages of levothyroxine therapy in delaying the onset of cardiovascular complications, this review makes the connection between subclinical hypothyroidism patients and the risk of cardiovascular complications-related death.

## Introduction and background

Hypothyroidism is a condition that occurs when serum thyroid-stimulating hormone (TSH) levels rise while free thyroxine or T4 (FT4) levels fall below the normal range. Triiodothyronine (T3) levels in the free state may be low or normal. Subclinical hypothyroidism is defined by serum TSH and T3 and T4 levels that are at the lower end of the normal range. Thyroid hormones regulate cardiac activity either directly or indirectly. T3 binds to the thyroid receptors in the myocardium and induces the passage of amino acids and calcium through the cardiac membrane. A decrease in free T3 and T4 reduces peripheral vascular resistance, increasing the afterload of the left ventricle [1,2]. Therefore, changes in thyroid hormone concentrations lead to respective cardiac dysfunction. This article reviews various studies and reports focusing primarily on cardiovascular complications due to subclinical hypothyroid conditions.

Functional anatomy and physiology of the thyroid gland

An endocrine gland, the thyroid, is situated in the front of the neck. It resembles a butterfly in shape. Histologically, the thyroid gland is composed of two types of C cells, namely, follicular and parafollicular. The thyroid hormones T3 and T4 are made from iodine and tyrosine in the colloids of follicular epithelial cells. This integration occurs on the surface of the thyroglobulin protein (Tg). Calcitonin is released by the gland’s parafollicular C cells. T3 is produced at relatively low levels, while T4 is the hormone the gland releases most frequently. T4 to T3 conversion occurs peripherally in numerous tissues, including the heart, liver, kidney, and muscles. Additionally, T4 can be transformed into the inactive metabolite reverse T3 (rT3).

**Figure 1:**
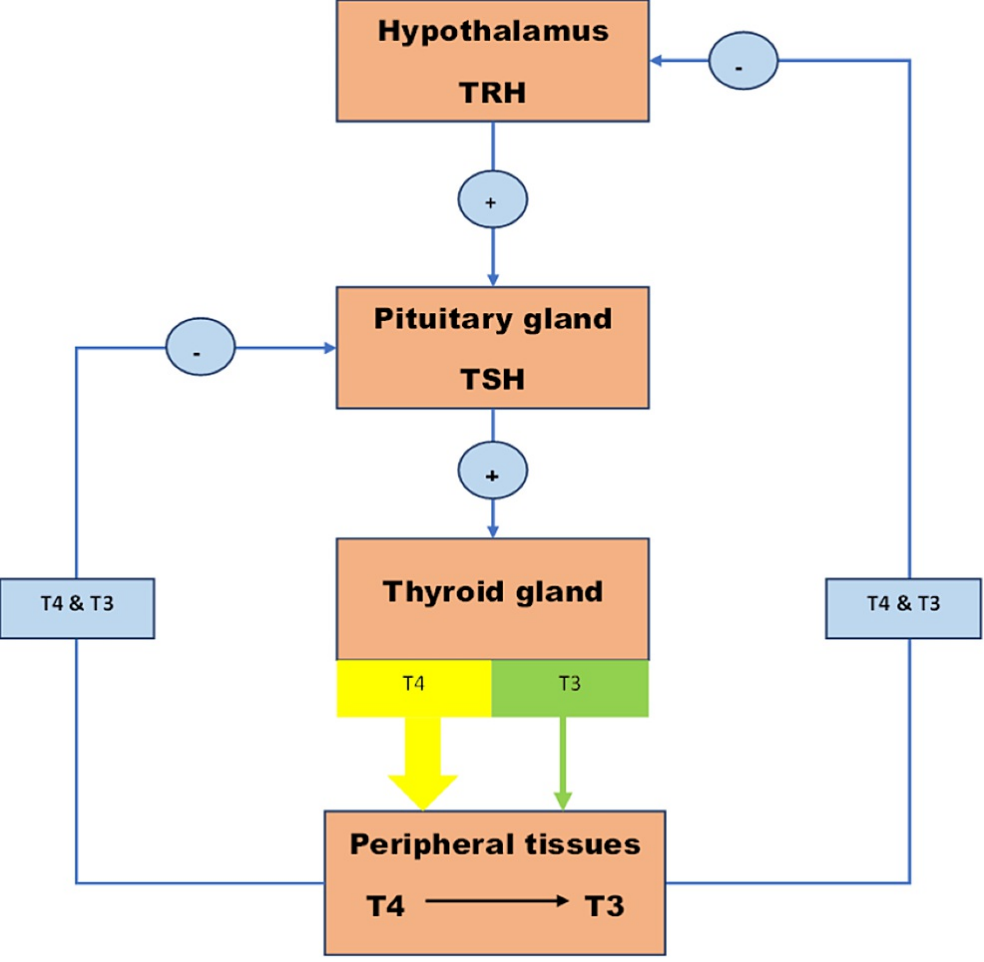
The hypothalamic-pituitary-thyroid axis. TRH = thyroid-releasing hormone; TSH = thyroid-stimulating hormone; T4 = thyroxine; T3 = triiodothyronine This image is authors’ own creation.

Thyrotropin, commonly known as TSH, is a glycoprotein made by the anterior pituitary that enhances the thyroid gland’s secretion of thyroid hormones. In reaction to the hypothalamus’s release of a thyroid-releasing hormone (TRH), TSH is secreted. According to Figure 1, thyroid hormone levels are a feedback regulator of TSH and TRH production. The hypothalamic-pituitary-thyroid axis goes through various complex physiological changes as we age [3].

Investigations of the thyroid gland

Thyroid disorders manifest as either oversecretion of hormones or insufficient circulating thyroid hormones. These disorders occur as a result of primary pathology within the gland or as a secondary disease that leads to abnormal stimulation of the thyroid gland. Investigations have revealed increased serum T3 and T4 levels and undetectable TSH as symptoms of thyrotoxicosis, also known as hyperthyroidism. The elevated blood TSH levels and decreased FT4 levels are signs of hypothyroidism. T3 levels in their free form can be increased or decreased. Serum TSH levels that are slightly elevated and serum T3 and T4 levels at the bottom of the reference range are characteristics of subclinical hypothyroidism.

In contrast, subclinical hyperthyroidism, or subclinical thyrotoxicosis, is seen with undetectable TSH but normal serum T3 and T4 concentrations. TSH is the most helpful investigation in thyroid function tests (TFT). Serum T3 is not a sensitive indicator of hypothyroidism; hence, only circulating T4 levels should be considered. TSH levels should be measured while fasting, whereas FT3/T4 has no relation to food. Any circulating antibodies concerning the thyroid should also be assessed. Antibodies such as thyroid peroxidase antibody (TPOAb), anti-thyroglobulin (TGAb), anti-TSH receptor (TSAb), and anti-microsomal antibodies are positive in autoimmune causes of thyroid disorders. TPOAbs have the maximum sensitivity for thyroid autoimmunity in subclinical hypothyroidism. They offer essential information on how quickly overt hypothyroidism develops in individuals with positive TPOAb compared to those with negative TPOAb [4]. Other investigations include radioisotope imaging, ultrasound, and fine-needle aspiration biopsy.

Subclinical hypothyroidism

Normal blood FT4 levels in the presence of elevated serum TSH levels are referred to as subclinical hypothyroidism. The incidence of subclinical hypothyroidism in the population under study ranges from 3% to 15%. Statistics indicate that subclinical hypothyroidism is more common in women and elderly adults [5]. The etiopathogenesis of subclinical hypothyroidism is the same as that of overt hypothyroidism. Autoimmunity (thyroiditis) is the most common cause of hypothyroidism. Other causes of primary hypothyroidism apart from autoimmunity include iodine deficiency and iatrogenic (radioactive iodine) [6]. Increasing age is an essential factor associated with decreased thyroid functioning in the elderly, which results in the overdiagnosis of subclinical hypothyroidism [7]. The female population is more predisposed to developing hypothyroidism or subclinical hypothyroidism, with the prevalence increasing with advancing age [4].

## Review

Methodology

We searched Medline via PubMed and Central databases via the Cochrane Library. The search strategy was tailored to individual databases and was as follows: Subclinical hypothyroidism OR cardiovascular complications OR thyroid therapy AND diagnosis. Furthermore, we screened the references list of potentially relevant studies. Studies retrieved from these electronic searches and relevant references included in the bibliography of those studies were reviewed (Figure 1).

**Figure 2:**
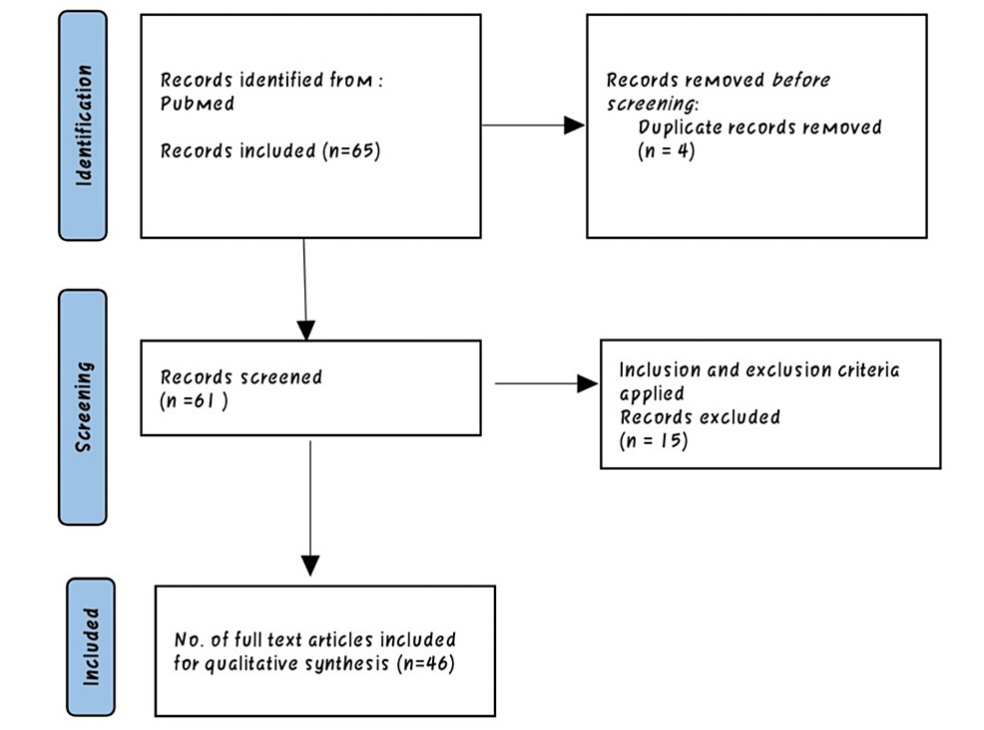
Preferred Reporting Items for Systematic Reviews and Meta-Analyses flowchart.

Clinical manifestations of subclinical hypothyroidism

The majority of the time, subclinical hypothyroidism has no symptoms. It may, however, exhibit hypothyroidism-related symptoms [8]. The following are the clinical signs of hypothyroidism: Integumentary symptoms: face puffiness, skin problems, hair loss, and loss of the outer third of the brows. Gastrointestinal symptoms: constipation, dysphagia, diminished appetite, excess weight, and cholelithiasis. Cardiovascular symptoms: bradycardia, pericardial effusions, and diastolic hypertension. Neurological symptoms: mononeuropathies, pseudo-dementia, and reduced attention. Musculoskeletal symptoms: weakness, cramping, stiffness, and weariness in the muscles. Reproductive symptoms: low libido, infertility, and irregular cycles [9]. An increased TSH with a normal T4 is the defining feature of subclinical hypothyroidism. According to recommendations, TSH and T4 should be tested at least once every two to three months. It is important to test TPO antibodies that point to an autoimmune cause of hypothyroidism. They are associated with a twofold elevated risk of subclinical hypothyroidism developing into overt hypothyroidism [10,11].

The action of thyroid hormones on the heart

Heart failure is a common occurrence in older individuals. Cardiovascular diseases are non-communicable diseases with significant morbidity and mortality rates. Recognizing the associated risk factors, modifiable and non-modifiable, is essential in healthcare. Subclinical thyroid dysfunction, hypo- or hyper-, is a potential contributor to developing cardiac complications in elderly individuals [12].

T3 is the biologically active form of the thyroid hormone. Free T3 and T4 decrease the peripheral vascular resistance (PVR), causing a decrease in diastolic blood pressure (DBP) and reducing the heart’s afterload. Thyroid hormones increase the sensitivity to circulating catecholamines resulting in increased heart rate and production. T3 enhances sympathetic nervous system activity by increasing the β-adrenergic receptors in the myocardium. Thyroid hormone receptors (TRs) in the myocardium regulate cardiac gene expression [13]. TR isoform (TRα1) is present in the heart, causing cardiac stimulation with a rise in the metabolic rate due to an increase in the heart rate and the force of contraction of the myocardium. Serum thyroid hormones, TRβ1, also play an essential (protective) role in cholesterol breakdown and activities of enzymes such as lipoprotein lipase and hepatic lipase, keeping a check on serum triglyceride levels [13]. It lowers plasma lipids, increases low-density lipoprotein (LDL) cholesterol uptake by the LDL receptor, and increases the basal metabolic rate. Additionally, the thyroid hormone is upregulated and downregulated by the sarcoplasmic reticulum calcium-ATPase 2 (SERCA2), Na+/K+ ATPase, and phospholamban. These modifications improve the heart’s ability to contract and relax during diastole [14].

Thyroid hormone dysfunction is partly responsible for the occurrence of heart diseases in apparently healthy individuals and augments any pre-existing heart condition. Subclinical hypothyroidism influences the development of cardiovascular disorders directly and indirectly. It contributes to various factors associated with cardiovascular diseases that collectively lead to impaired heart function. Subclinical hypothyroidism increases the incidence of heart failure when the thyrotrophin levels are more than 10mIU/L (normal range = 0.5mIU/L-5.0 mIU/L) [15].

Subclinical hypothyroidism and the heart

Age-related declines in thyroid hormone clearance are accompanied by reductions in thyroid hormone secretion, which maintains total and FT4 levels in the serum. Unlike thyroxine, serum total and FT3 fall as people age. This decrease is thought to be caused mainly by decreased peripheral T4 to T3 conversion, which may directly result from nonthyroidal sickness or simply aging. T4 is one of the most significant regulators for monitoring cardiac activity and cardiovascular hemodynamics [16]. Impaired thyroid hormone levels affect the relaxing capacity of vascular muscles and reduce the contractile capability of the myocardium by controlling calcium uptake and the heart’s ability to pump blood [17,18].

Previous studies found that subclinical hypothyroid individuals had systolic dysfunction after exertion and diastolic dysfunction at rest and that restoring euthyroidism cured these anomalies. Normalization of heart function with euthyroidism resulted in a decrease in isovolumic relaxation time, a decline in pre-ejection period/ejection time, an increase in the early diastolic/late diastolic mitral flow velocity ratio, and a rise in the left ventricular ejection fraction ratio [19,20]. Age-related diseases such as atherosclerosis, coronary heart disease, and neurological conditions are affected by intricate physiological changes in the hypothalamic-pituitary-thyroid axis [3]. Traditional cardiovascular risk factors, such as a rise in homocysteine and low-density lipoprotein-cholesterol (LDL-C), as well as the emergence of a procoagulant state, are aggravated by thyroid failure [21,22]. Cardiovascular disease was more common in men with subclinical hypothyroidism under 50 compared to men with euthyroidism [22]. The most common cardiovascular diseases with subclinical hypothyroidism include coronary artery disease, arterial hypertension, hypercoagulation, atrial fibrillation, and heart failure.

Coronary Artery Disease

The thyroid hormone affects lipids primarily by increasing the receptors for LDL at the level of the liver and periphery and by increasing the action of the transcription factor sterol regulatory element-binding protein 2 (SREBP-2), which positively modulates the activity of LDL receptors. T4 controls the function of the 3-hydroxy-3-methylglutaryl-coenzyme A (HMG-CoA) enzyme’s rapid degradation by influencing the expression of the *SREBP-2* gene [23]. It was recently discovered that subclinical hypothyroidism patients have higher serum levels of apolipoprotein B-48, a component of chylomicrons remnants and chylomicron and a putative predisposing determinant for atherosclerosis [24].

Arterial Hypertension

An arterial high blood pressure is characterized by pressure indicators equivalent to or more than 140 mmHg for the systolic pressure or 90 mmHg for the diastolic pressure after repeatedly assessing the arterial tension. In an analysis of the connection between subclinical hypothyroidism and systolic blood pressure (SBP), subclinical hypothyroidism was associated with increased systolic blood pressure. Therefore, it was hypothesized that it can act as a potential risk factor for increased systolic blood pressure [25]. Vascular stiffness and malfunction of the left ventricle’s diastolic chamber have also been linked to hypothyroidism [7,18].

Hypercoagulation

Recently, subclinical hypothyroidism has been linked to elevated C-reactive protein levels, homocysteine, raised arterial stiffness, endothelial disruption, and altered coagulation parameters [26]. Hypercoagulable and hypofibrinolytic states may be important factors in the progress of atherosclerosis in subclinical hypothyroidism individuals, according to reports of abnormalities in hemostatic factors. The latter is primarily directed by the overactivity of plasminogen activator inhibitor-1 and factor VII [27].

Heart Failure

A lack of thyroid hormone may increase the risk of heart failure incidents. Subclinical hypothyroidism may exacerbate the aging-related cardiac changes that raise the risk of heart failure development and events [28]. A population-based study revealed that patients tracked for four years with a TSH level of 7 mIU/L or above had an increased predisposition to heart attacks than euthyroid people [29,30]. There was a statistically substantial threat of heart failure amongst individuals with a TSH level of 10 mIU/L. The probability of heart failure incidents was enhanced in individuals with higher TSH levels compared to control values [31]. According to several kinds of research, subclinical hypothyroidism may put individuals with chronic heart failure at risk for cardiac mortality [32,33].

Management

Most patients with subclinical hypothyroidism do not require any specific treatment as they do not always present with significant symptoms [4]. Patients with subclinical hypothyroidism are treated symptomatically at any given point rather than starting levothyroxine therapy immediately. Management of the thyroid disorder and the comorbid complication so developed is essential. Individuals with subclinical hypothyroidism are categorized into two categories based on their serum TSH levels to aid their treatment: Mild subclinical hypothyroidism: individuals in whom TSH levels are between 5.0 and 10.0 mIU/L. Severe subclinical hypothyroidism: individuals in whom TSH levels are above 10.0 mIU/L. Indications for subclinical hypothyroidism treatment include symptomatic, TSH > 10 mIU/L, pregnant women, and positive antibodies. Mild subclinical hypothyroidism individuals should be monitored closely, and regular follow-ups should be conducted to keep the progression of the disease or any other associated complications in check. Worsening of the symptoms is an immediate indication to start the individual on appropriate therapy [25]. Moderate or severe subclinical hypothyroidism individuals may require immediate L-thyroxine therapy as per their condition. Elderly patients (>65 years) generally require treatment and regular evaluation.

The goal is to normalize the TSH levels regardless of the cause of hypothyroidism. Levothyroxine is the treatment of choice. Treatment is usually initiated with a once-daily dose of levothyroxine sodium (1.6 µg/kg/day or 100 µg/day). Start with a low dose of 50 µg/day for three weeks, thereafter increasing to 100 μg/day for the next three weeks, and, finally, a dose of 100-150 μg/day should be maintained. TSH levels should be monitored at six to eight weeks after the initiation of the therapy or dosage change. In the elderly, treatment should be started with a half dose with a gradual increase. The dosage must be adjusted as per the requirement of individual patients.

The combined therapy of L-thyroxine with L-triiodothyronine is another modality in the treatment of subclinical hypothyroidism and hypothyroidism. However, its efficacy or advantage over L-thyroxine monotherapy is still unproven [9]. Levothyroxine (L-thyroxine) is the treatment of choice in subclinical hypothyroidism. Starting an individual on the recommended full dose of L-thyroxine right away is the most effective and secure course of action. Patients with existing ischemic heart disease are an exception to this rule and should begin treatment with lower doses before gradually increasing the dosage [34]. Long-term therapy with thyroxine can protect the heart from ischemia [35]. Whether levothyroxine has the potential to lower cardiovascular disease risk in people with subclinical hypothyroidism is still unknown [13].

## Conclusions

Females in their reproductive years and the elderly are more likely to have subclinical hypothyroidism. Though asymptomatic, it requires investigation, diagnosis, and regular screening. The most common complication associated is dyslipidemia. The higher risk of congestive heart disease mortality associated with subclinical hyperthyroidism has been attributed to the systemic effects of thyroid hormone, such as a change in heart function or cardiac arrhythmia. Screening for cardiovascular risk factors did not change the odds of subclinical hyperthyroidism supports these views. Evidence suggests that people with plasma TSH levels above 10 mIU/L may be at greater risk of cardiovascular disease. Higher TSH levels are associated with higher congestive heart disease morbidity and mortality. There is a risk in the initiation of levothyroxine treatment in elderly patients such as cardiovascular complications. Furthermore, there is currently little agreement on the clinical relevance and treatment of this moderate form of thyroid insufficiency.
